# Assessment data of antibiotic resistance patterns in bacteria from Ana Sagar Lake: Environmental and public health perspectives

**DOI:** 10.1016/j.dib.2026.113150

**Published:** 2026-08-10

**Authors:** Ayushi Jain, Sanju Kumari, Khem Raj Meena, Jitendra, Arvind Kumar

**Affiliations:** a Department of Biotechnology, School of Life Sciences, Central University of Rajasthan, 305817, India; b Department of Biotechnology, Maharaja Surajmal Brij University, Bharatpur, India

**Keywords:** Antibiotics, Ana Sagar Lake, Antibiotic resistance, Environment

## Abstract

Ana Sagar Lake, despite its ecological, public health, and economic importance, has remained largely unexplored with respect to its bacterial diversity and antibiotic resistance. Therefore, the present study aimed to characterize the bacterial diversity of the lake and evaluate the antibiotic resistance profiles of the recovered isolates. Based on 16S rRNA gene sequencing, the bacterial isolates AL-1, AL-5, AL-6, and AL-9 were identified as *Priestia* sp. (GenBank accession: PX973463), *Acinetobacter soli* strain AL-5 (PX973464), *Pseudomonas* sp. AL-6 (PX973465), and *Acinetobacter* sp. AL-9 (PX973466), respectively. Among these, AL-1 (*Priestia* sp.) exhibited the highest level of resistance, showing resistance to chloramphenicol, streptomycin, cefepime, aztreonam, and cefoperazone. Interestingly, AL-9 demonstrated an inverse response to antibiotic exposure, with significantly enhanced biofilm formation in the presence of chloramphenicol compared with the untreated control. In addition, AL-1, AL-6, and AL-9 displayed increased intracellular accumulation of ethidium bromide (EtBr), indicating reduced efflux pump activity and a potential increase in antibiotic susceptibility. Chloramphenicol exerted the most pronounced effect on AL-9 cells among the antibiotics tested. To the best of our knowledge, this is the first study to characterize the antibiotic resistance profiles of bacterial isolates recovered from Ana Sagar Lake. These findings provide valuable baseline data for assessing the microbial quality of the lake, understanding the potential public and environmental health risks associated with its use, and supporting future monitoring and management strategies.

Specifications Table

**Table:** 

Subject	Microbiology, Bacteriology, antimicrobial resistance
Specific subject area	Antimicrobial resistance, Ana Sagar Lake, bacterial diversity
Data format	Analyzed,
Type of data	Tables, figures
Data collection	A water sample was collected from Ana Sagar Lake in Ajmer, Rajasthan. At each site, 50 mL of surface water (∼30 cm depth of the surface) was collected in sterile polypropylene bottles while wearing gloves. These polypropylene bottles were pre-sterilized by autoclaving at 121 °C for 15 min. During collection, aseptic conditions were maintained to prevent external contamination. The sampling collection tubes were marked with location, date, time, and sample name. Sample was delivered to the lab as soon as possible on a cold ice bag to protect them from ambient temperatures. Samples were transported to the laboratory in an insulated ice box (4 °C) and processed within 4–6 h of collection for bacterial isolation on different media. All bacteria were assessed for their physicochemical and biochemical properties, and antimicrobial resistance was analyzed by the Kirby-Bauer disk diffusion method.
Data source location	Site: Ana Sagar lake Country: India State: Rajasthan Dist.: Ajmer Coordinates: (Latitude: 26.480357 / N 26° 28´ 49.284˝ and Longitude: 74.628897 / E 74° 37´ 44.028˝)
Data accessibility	All four bacterial isolates (AL-1, AL-5, AL-6, AL-9) sequences submitted to NCBI and their link for details are given below; https://www.ncbi.nlm.nih.gov/nuccore/PX973463.1/ https://www.ncbi.nlm.nih.gov/nuccore/PX973464.1/ https://www.ncbi.nlm.nih.gov/nuccore/PX973465.1/ https://www.ncbi.nlm.nih.gov/nuccore/PX973466.1/
Related research article	Sonkar, V., Devtalla, H. & Kumar, S. Pesticide-driven antimicrobial resistance in water bodies: insights on environmental concerns, health implications and mitigation strategies. *Environ Geochem Health* 47, 282 (2025). https://doi.org/10.1007/s10653-025-02600-y

## 1 Value of this Data

• To our best knowledge, this is the first microbiological study on Lake Ana Sagar, India.
• This study is more valuable in terms of potential consequences and associated microbial risks in lake water, if any, because one of the biggest global public health problems of the 21st century is antimicrobial resistance (AMR).
• This study provides comprehensive and reliable data on bacterial diversity, antibiotic resistance profiles, and biofilm-forming capabilities of isolated bacteria.
• The findings of this study may serve as a valuable resource for further investigations into bacterial ecology, bioprospecting potential, pathogenicity assessment, and the development of effective strategies for sustainable water use and management.

## 2 Background

Ana Sagar Lake is one of the principal freshwater bodies in Ajmer district, Rajasthan, India. It spans 13 km with a 47,50,000 m^3^ water volume with a maximum depth of 14 Ft and has geographic location (Latitude- 26°25’N-26°29’N and Longitude −74°38’E-74°42’E). The emergence of AMR in microbes is driven by the overuse of antibiotics available in the market [1,2]. Bacteria develop resistance through several mechanisms, including limiting antibiotic uptake, modifying drug targets, enzymatic inactivation of antibiotics, and actively expelling antibiotics via efflux pumps [3]. The rising burden of AMR contributes to healthcare costs by prolonging hospital stays and increasing treatment costs [4]. Beyond its clinical implications, the dissemination of antibiotic-resistant bacteria and resistance genes into aquatic ecosystems threatens ecological balance, adversely affecting microbial communities, aquatic flora and fauna, and overall water quality.

The occurrence and persistence of antimicrobial-resistant (AMR) bacteria in Ana Sagar Lake are likely driven by the combined influence of anthropogenic activities and environmental factors. Urban lakes receiving untreated or partially treated waste water, sewage leakage, agricultural effluents, and livestock waste are recognized as important reservoirs of antibiotic residues, resistant microorganisms, and mobile genetic elements, all of which contribute to the maintenance of selective pressure and the dissemination of antimicrobial resistance [5]. Additionally, fecal contamination originating from domestic animals, wild birds, and human recreational and domestic activities surrounding the lake may further enrich the environmental resistome through the continuous introduction of resistant bacteria. Environmental conditions, including seasonal rainfall, temperature fluctuations, nutrient enrichment (eutrophication), and sediment–water interactions, can enhance the persistence, proliferation, and horizontal transfer of antimicrobial resistance genes among microbial communities. Collectively, these factors suggest that Ana Sagar Lake functions as a dynamic environmental reservoir and dissemination hotspot for AMR bacteria, where fecal pollution, ecological selection, and horizontal gene transfer interact to sustain and propagate antimicrobial resistance within the aquatic ecosystem [6,5]. The findings of this study may serve as valuable data for further investigations into bacterial ecology, bioprospecting potential, pathogenicity assessment, and the development of effective strategies for sustainable water use and its management. Future research should focus on developing reliable, cost-effective strategies for antibiotic removal, and advanced oxidation processes/remediation methods are suggested for greater research attention [6].

## 3 Data Description

In this study, bacterial diversity and antibiotic resistance profiles were investigated using culturable bacterial isolates recovered from Ana Sagar Lake. A total of 13 bacterial isolates (AL-1 to AL-13) were isolated on different culture media, including Nutrient Agar (NA), Eosin Methylene Blue (EMB) agar, and MacConkey agar. The purified isolates were characterized based on their colony morphology, Gram reaction, and biochemical properties, and the results are summarized in Table 1. The biochemical characterization revealed that all isolates were Gram-negative except isolate AL-3, which was Gram-positive. Starch hydrolysis was observed in isolates AL-1, AL-3, AL-6, AL-7, and AL-13, indicating their ability to produce extracellular amylase, whereas the remaining isolates were unable to hydrolyze starch. Indole production was detected only in isolates AL-2 and AL-3, suggesting the presence of the enzyme tryptophanase responsible for the conversion of tryptophan into indole. The remaining isolates were indole-negative, indicating the absence of tryptophanase activity. All 13 bacterial isolates tested positive for the catalase assay, demonstrating their ability to produce the catalase enzyme, which detoxifies hydrogen peroxide and protects cells from oxidative stress.

**Table 1:** Morphological and Gram-staining characteristics of purified bacterial isolates.

Media	Bacterial Isolate	Colony Colour	Colony Shape & Size	Morphology	Gram’s characteristics	Biochemical tests		
						Starch hydrolysis	Indole test	Catalase test
NA	AL- 1	Yellow	Round & Small	streptobacillus	Gram -ve	**P**	N	**P**
	AL- 2	Orangish red	Regular & Small	streptococcus	Gram -ve	N	**P**	**P**
	AL- 3	Yellow creamy	Regular & Medium	coccus	Gram +ve	**P**	**P**	**P**
	AL- 4	White	Regular & small	monobacillus	Gram -ve	N	N	**P**
	AL- 5	White	Bulgy & medium	coccobacillus	Gram -ve	N	N	**P**
EMB	AL- 6	Transparent	Patchy & large	bacillus	Gram -ve	**P**	N	**P**
	AL- 7	Transparent	Patchy & medium	monobacillus	Gram -ve	**P**	N	**P**
	AL- 8	Pink	Circular & small	irregular coccus	Gram -ve	N	N	**P**
	AL- 9	White	Circular & small	coccobacillus	Gram -ve	N	N	**P**
Mac-conkey	AL- 10	Transparent with pink	Regular	Irregular bacillus	Gram -ve	N	N	**P**
	AL- 11	White	Regular & small	monococcus	Gram -ve	N	N	**P**
	AL- 12	Pink	Regular & small	diplococcus	Gram -ve	N	N	**P**
	AL- 13	Transparent	Regular & medium	monococcus	Gram -ve	**P**	N	**P**

p: positive; N: negative.

All bacterial isolates were subsequently evaluated for their antibiotic susceptibility using the Kirby–Bauer disk diffusion method. Briefly, bacterial cultures were uniformly spread on Mueller–Hinton agar (MHA) plates, and antibiotic discs were placed on the inoculated surface. Following incubation, the diameter of the zone of inhibition surrounding each antibiotic disc was measured to determine the susceptibility of the isolates to the tested antibiotics. Among the 13 isolates, four (AL-1, AL-5, AL-6, and AL-9) were identified as multidrug-resistant (MDR), as they exhibited resistance to three or more antibiotics. Isolate AL-1 showed the highest level of resistance, being resistant to five antibiotics, namely chloramphenicol, streptomycin, cefepime, aztreonam, and cefoperazone (Table 2). Isolate AL-5 was resistant to chloramphenicol, penicillin, and streptomycin, while exhibiting intermediate susceptibility to amikacin. Isolate AL-6 displayed resistance to clindamycin, chloramphenicol, cefepime, and aztreonam. Similarly, isolate AL-9 was resistant to clindamycin, penicillin, and vancomycin but remained susceptible to the other antibiotics tested.

**Table 2:** Antibiotic susceptibility testing of bacterial isolates.

Antibiotics	Bacterial Isolate			
	AL-1	AL-5	AL-6	AL-9
Tetracycline (TE)	10 mm	11 mm	10 mm	8 mm
Clindamycin (CD)	4 mm	8 mm	**R**	**R**
Amikacin (AK)	5 mm	4 mm	8 mm	9 mm
Chloramphenicol (CL)	**R**	**R**	**R**	3 mm
Meropenem (MRP)	15 mm	16 mm	12 mm	16 mm
Imipenem (IPM)	19 mm	-	13 mm	14 mm
Penicillin (P)	15 mm	**R**	9 mm	**R**
Streptomycin (S)	**R**	**R**	6 mm	6 mm
Ampicillin (AMP)	14 mm	12 mm	7 mm	6 mm
Cotrimoxazole (COT)	13 mm	13 mm	14 mm	6 mm
Vancomycin (VA)	7 mm	10 mm	7 mm	**R**
Cefepime (CPM)	**R**	14 mm	**R**	11 mm
Aztreonam (AT)	**R**	16 mm	**R**	7 mm
Cefoperazone (CFP)	**R**	12 mm	2 mm	12 mm
Azithromycin (AZM)	5 mm	9 mm	8 mm	8 mm

All four isolates (AL-1, AL-5, AL-6, and AL-9) were resistant to chloramphenicol in the Kirby–Bauer disk diffusion assay. However, the biofilm assay demonstrated a progressive reduction in biofilm viability with increasing chloramphenicol concentrations. This apparent discrepancy is expected because the disk diffusion method assesses bacterial susceptibility to a fixed antibiotic concentration, whereas the biofilm assay evaluates the response of pre-established biofilms to a range of antibiotic concentrations. In the present study, chloramphenicol concentrations of 10–30 μg/ml progressively reduced biofilm viability, indicating that higher antibiotic concentrations can partially overcome biofilm-associated tolerance and impair bacterial cell viability.

Based on colony morphology, pigmentation, cell morphology, and antibiotic resistance profiles, four bacterial isolates (AL-1, AL-5, AL-6, and AL-9) were selected for further characterization. The isolates were identified by Sanger sequencing of the 16S rRNA gene using the universal primer pair 27F and 1492R. PCR amplification generated an approximately 1500 bp amplicon, which was resolved on a 1% agarose gel containing ethidium bromide N

**Fig. 1:**
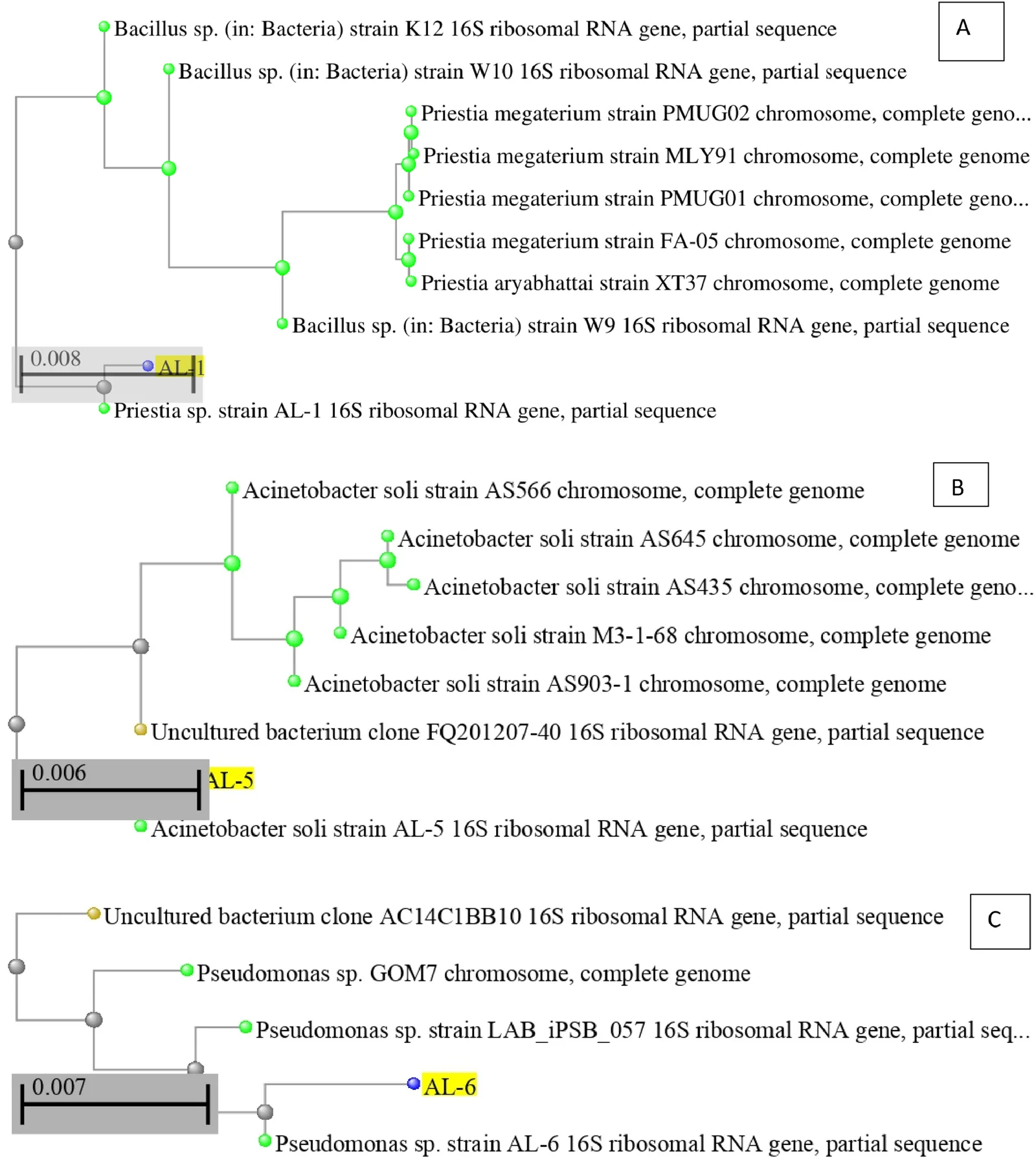

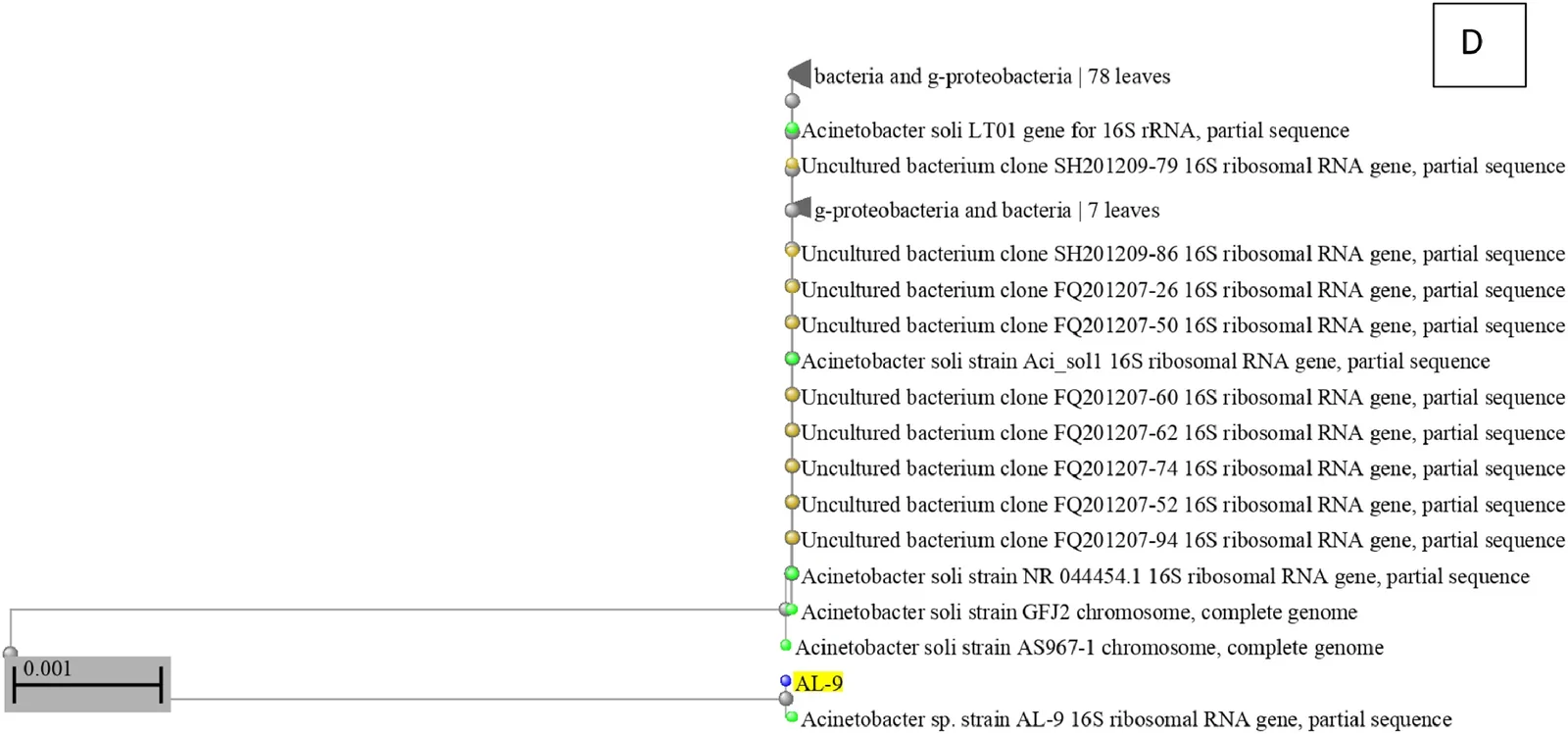
Phylogenetic tree of bacteria. **(A)** Bacteria isolate AL-1; (B) bacterial isolate AL-5; (C) bacteria isolate AL-6; and (D) bacterial isolate AL-9, were found as *Priestia* sp*., Acinetobacter soli, Pseudomonas* sp., *Acinetobacter* sp., respectively.

N(EtBr) and visualized using a Bio-Rad Gel Documentation System (Supplementary Figure 1). The expected amplicon size (∼1500 bp) was confirmed using a Takara DNA ladder. The obtained forward and reverse sequences were assembled and analyzed using BLASTN, followed by phylogenetic analysis (Fig. 1). Sequence analysis identified isolates AL-1, AL-5, AL-6, and AL-9 as *Priestia* sp., *Acinetobacter soli* strain AL-5, *Pseudomonas* sp. AL-6, and *Acinetobacter* sp. AL-9, respectively.

**Fig. 2:**
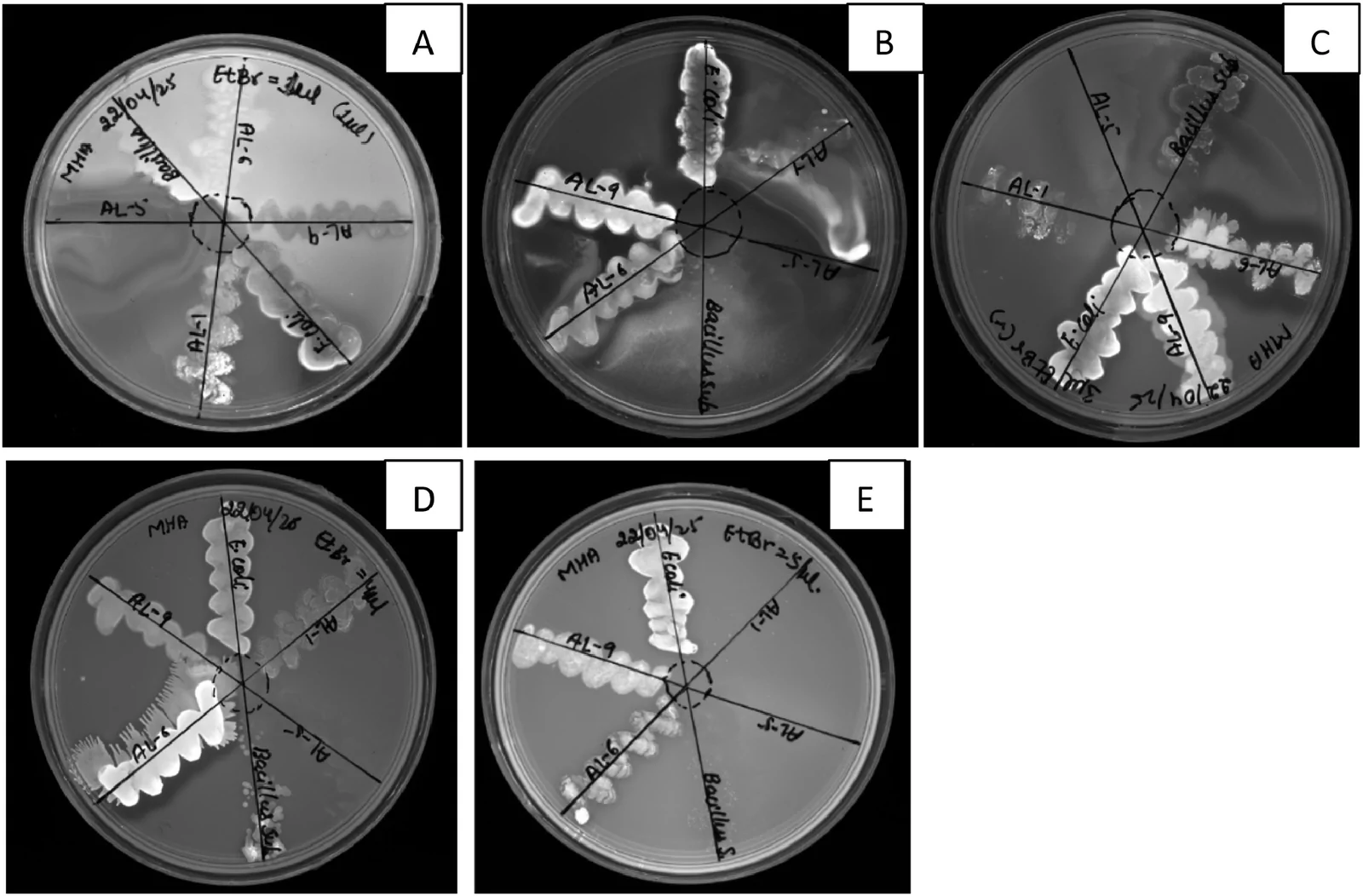
A-E; Cartwheel test performed in different EtBr concentrations at agar plates.

Ethidium bromide (EtBr) concentrations ranging from 1 to 5 µg/mL were used to evaluate the fluorescence patterns of the bacterial isolates. Among the tested isolates, AL-1 exhibited weak fluorescence, indicating limited intracellular accumulation of EtBr and suggesting moderate efflux pump activity. AL-5 showed no detectable fluorescence at any of the tested EtBr concentrations, indicating minimal intracellular EtBr accumulation and suggesting the highest efflux activity among the isolates. In contrast, AL-6 and AL-9 displayed strong fluorescence, reflecting substantial intracellular accumulation of EtBr and indicating relatively low or inactive efflux pump activity (Fig. 2).

**Fig. 3:**
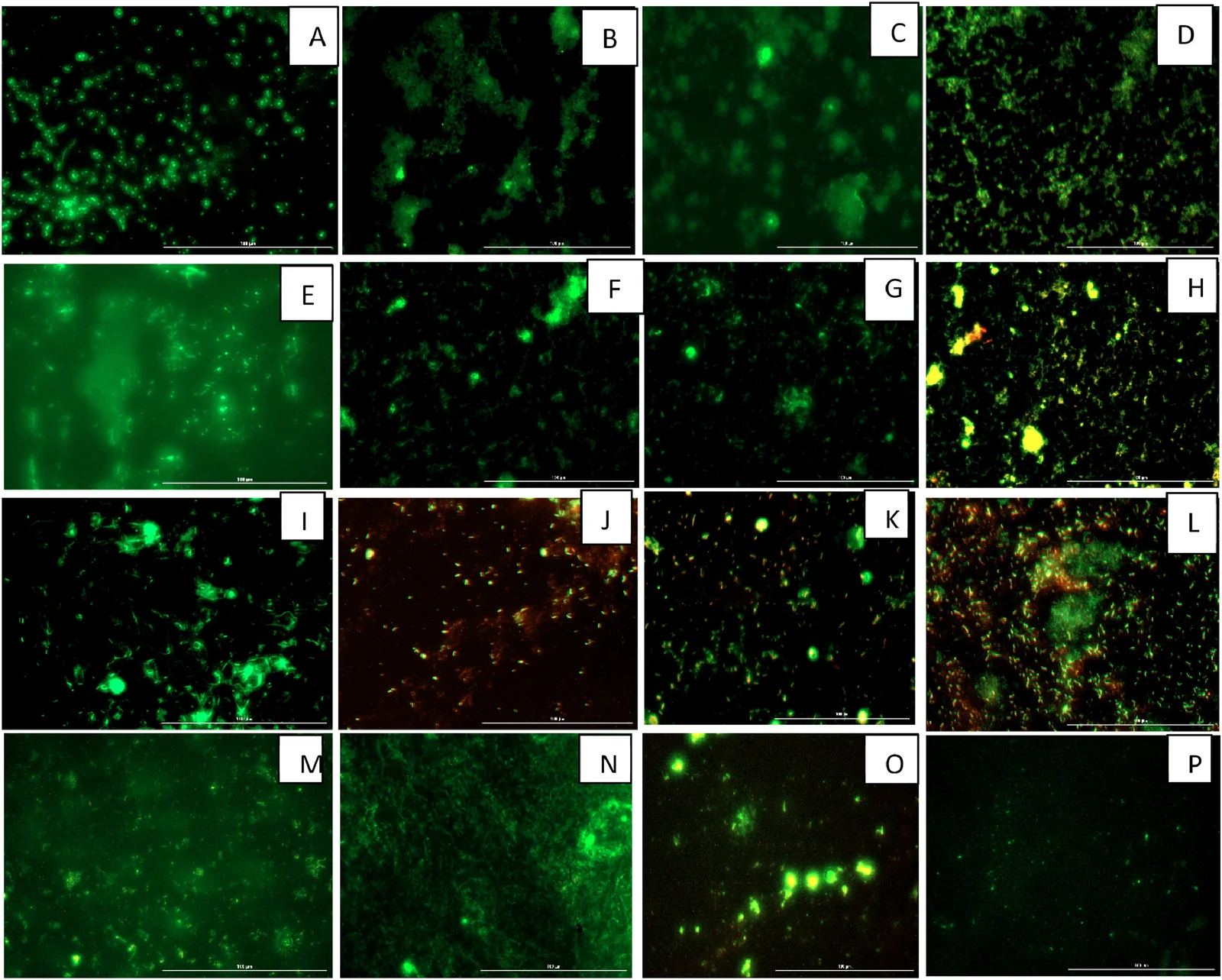
Effect of chloramphenicol on bacteria. (A-D) *Priestia* sp. (PX973463); Control (Biofilm-forming live cells without chloramphenicol treatment), 10 µg/ml chloramphenicol, 20 µg/ml chloramphenicol, 30 µg/ml chloramphenicol respectively. **(E-H)***Acinetobacter soli* strain AL-5 (PX973464); Control (Biofilm-forming live cells without chloramphenicol treatment), 10 µg/ml chloramphenicol, 20 µg/ml chloramphenicol, 30 µg/ml chloramphenicol respectively. **(I-L)***Pseudomonas* sp. AL-6 (PX973465); Control (Biofilm-forming live cells without chloramphenicol treatment), 10 µg/ml chloramphenicol, 20 µg/ml chloramphenicol, 30 µg/ml chloramphenicol respectively. **(M-P)***Acinetobacter* sp. (PX973466); Control (Biofilm-forming live cells without chloramphenicol treatment), 10 µg/ml chloramphenicol, 20 µg/ml chloramphenicol, 30 µg/ml chloramphenicol respectively.

The antibacterial activity of chloramphenicol against biofilm-forming bacterial isolates demonstrated a concentration-dependent response (Fig. 3). In isolates AL-1 and AL-5, treatment with 10 and 20 µg/ml chloramphenicol did not significantly reduce biofilm biomass or bacterial viability, indicating resistance at these concentrations. However, exposure to 30 µg/ml resulted in a marked reduction in viable cells, suggesting that a higher chloramphenicol concentration was required to overcome biofilm-associated tolerance and exert a bactericidal effect. Similarly, the AL-6 isolate exhibited minimal inhibition at 10 µg/ml, followed by progressively greater reductions in bacterial viability at 20 and 30 µg/ml, indicating partial susceptibility at elevated antibiotic concentrations. For isolate AL-9, treatment with 10 µg/ml produced no significant effect on biofilm biomass or viability, whereas 20 µg/ml caused moderate bacterial cell death and 30 µg/ml resulted in a substantial reduction in viable cells, demonstrating concentration-dependent susceptibility to chloramphenicol. The observed differences between the disk diffusion and biofilm assays can be attributed to the experimental design, as the disk diffusion method evaluates bacterial susceptibility using a single fixed antibiotic concentration, whereas the biofilm assay assesses the effects of multiple chloramphenicol concentrations on established biofilms.

## 4 Experimental Design, Materials and Methods

### 4.1 Collection of water sample and isolation of bacteria

The surface water sample (Latitude: 26.480357 / N 26° 28´ 49.284˝ and Longitude: 74.628897 / E 74° 37´ 44.028˝) of Ana Sagar Lake (Ajmer) was collected in an autoclaved polypropylene bottle as reported by [7]. The water sample was diluted serially (10^−1^ to 10^−10^), and an aliquot of 100 µl of diluted water sample (10^−3^, 10^−5^, 10^−7^ & 10^−9^) was spread over the agar plates prepared from Nutrient Agar (NA), Eosin Methylene Blue (EMB), and MacConkey agar media. The plates were labelled and sealed with parafilm, then incubated at 37 °C for 24 h [8]. All the morphologically different isolated colonies enumerated on culture media plates (Nutrient agar (NA), Mac-Conkey and Eosin Methylene Blue (EMB) agar media plates), were picked and purified by the streak plate method on fresh agar plates of respective media and further were preserved at 4 °C refrigerator [8].

### 4.2 Antibiotic susceptibility tests of bacteria

100 µl (1 × 10^5^cells) of freshly grown 6 h old culture in TSB broth was spread evenly onto Mueller-Hinton Agar (MHA) plates using a pre-sterilized glass spreader. The plates were allowed to dry for 5 min in aseptic conditions, and antibiotic rings (were placed gently over the spread culture under sterile conditions. The plates were sealed with parafilm and incubated at 37 °C overnight. Zone of inhibition was observed after 24 h to check susceptibility and resistance to a specific antibiotic, and results were interpreted as described previously [9]. Experiment was performed in the triplicates to lower down the error of the zone of clearance.

### 4.3 *DNA isolation and molecular characterization of bacterial isolates by 16s rRNA sequencing*

Bacterial cultures (AL-1, AL-5, AL-6, AL-9) were grown overnight in LB broth to an OD of 1.0, and whole-cell DNA was extracted as described earlier. Whole cell DNA was taken as a template to amplify 16S rDNA coding rRNA by using primer pairs 27F and 1492R and run on agarose gel (1%). The amplified PCR product was sequenced at Barcode Biosciences pvt. Ltd (Bangalore) through Sanger sequencing. Sequence analysis was performed manually and using the BlastN online tool available at NCBI (http://www.ncbi.nlm.nih.gov/). The 16S rDNA sequences of closely related taxa were retrieved from the database, and multiple sequence analysis was performed using the ClustalW tool embedded in MEGA11. The phylogenetic tree was constructed in MEGA 11 using the Neighbor-Joining method [10,11].

### 4.4 Cartwheel assay for assessment of efflux pump activity in bacterial isolates

To assess efflux pump activity in bacteria, growth was monitored until the optical density (OD₆₀₀) reached approximately 1.0 in Mueller-Hinton broth (MHB). MHA media was prepared by adding different volumes of ethidium bromide (EtBr, stock concentration 10 mg/mL) to each plate such as 1 µL, 2 µL, 3 µL, 4 µL, and 5 µL. A sterile cotton swab bacterial cultures in a radial, cartwheel pattern on the Petri plate. The swab was dipped into each bacterial suspension—namely AL-1, AL-5, AL-6, and AL-9. Starting from the centre of the agar surface, the swab was streaked outward toward the edge of the plate to form a spoke-like design [12].

### 4.5 Anti-adhesive activity of chloramphenicol against biofilm-forming bacterial strains

Biofilm-forming activity of bacteria was assessed as reported by [13]. Each isolate was then treated with chloramphenicol at three different concentrations (10 µg/ml, 20 µg/ml, and 30 µg/ml) by adding 300 μl of the respective antibiotic solution to the 12 well plate, while one well per isolate served as a control of 1**×** PBS [11]. The plate was incubated for 2 h at 37 °C. After treatment, the wells were gently washed with PBS, and to evaluate biofilm viability and adherence, each well was stained with 5 µl of a pre-mixed dye solution containing FDA (fluorescein diacetate) and EtBr (ethidium bromide), followed by incubation for 5 min at room temperature in the dark and thereafter observed under a multimode fluorescence reader at 10X magnification to visualize and differentiate live (green fluorescence) and dead (red fluorescence) cells. This experiment was performed in the triplicates.

## Limitations

Not applicable.

## CRediT Author Statement

**Ayushi Jain:** Conceptualization, Data curation. **Khem Raj Meena:** Writing – review & editing, Methodology, Investigation. **Sanju Kamari:** Investigation. **Jitendra:** Investigation. methodology, Visualization. **Arvind Kumar:** Data analysis.

## Ethics Statement

Authors have read and followed the ethical requirements for publication in Data in Brief and confirmed that the current work does not involve human subjects, animal experiments, or any data collected from social media platforms.

## Data Availability

The Sanger sequencing project of 04 bacterial isolates was deposited in NCBI GenBank and the link given below;

https://www.ncbi.nlm.nih.gov/nuccore/PX973463.1/

https://www.ncbi.nlm.nih.gov/nuccore/PX973464.1/

https://www.ncbi.nlm.nih.gov/nuccore/PX973465.1/

https://www.ncbi.nlm.nih.gov/nuccore/PX973466.1/ and got accession number PX973463, PX973464, PX973465, PX973466 for bacteria.

## Appendix Supplementary materials

Image, application 1
