# Supplementary material for: Assessment data of antibiotic resistance patterns in bacteria from Ana Sagar Lake: Environmental and public health perspectives
Source: Data Brief. 2026 Aug 10;68:113150. doi: 10.1016/j.dib.2026.113150 (PMC13519263; doi:10.1016/j.dib.2026.113150)
Supplement: Supplementary file 1 [file mmc1.docx]

**Figure S1: Agarose gel analysis for 16S rRNA PCR amplification. A – Agarose gel analysis for PCR product; Lane 1: DNA ladder and Lane 2-8: PCR product of different bacterial isolates and B – Agarose gel analysis for PCR clean-up Amplicons**

**Forward and Reverse sequences of bacterial isolates**

AL-1

>0424_894_011_PCR_AL_1_27F_E10.ab1

AAACCTGGGGCGGCGATGACGTGATACATGCAAGTCGAGACGAACGTGATCTAGTAAGCTTGCTTCTATGACGTTAGCGG

CGGACGGGTGAGTAACACGTGGGCAACCTGCCTGTAAGACTGGGATAACTTCGGGAAACCGAAGCTAATACCGGATAGGA

TCTTCTCCTTCATGGGAGATGATTGAAAGATGGTTTCGGCTATCACTTACAGATGGGCCCGCGGTGCATTAGCTAGTTGG

TGAGGTAACGGCTCACCAAGGCAACGATGCATAGCCGACCTGAGAGGGTGATCGGCCACACTGGGACTGAGACACGGCCC

AGACTCCTACGGGAGGCAGCAGTAGGGAATCTTCCGCAATGGACGAAAGTCTGACGGAGCAACGCCGCGTGAGTGATGAA

GGCTTTCGGGTCGTAAAACTCTGTTGTTAGGGAAGAACAAGTACAAGAGTAACTGCTCGTACCTTGACGGTACCTAACCA

GAAAGCCACGGCTAACTACGTGCCAGCAGCCGCGGTAATACGTAGGTGGCAAGCGTTATCCGGAATTATTGGGCGTAAAG

CGCGCGCAGGCGGTTTCTTAAGTCTGATGTGAAAGCCCACGGCTCAACCGTGGAGGGTCATTGGAAACTGGGGAACTTGA

GTGCAGAAGAGAAGAGCGGAATTCCACGTGTAGCGGTGAAATGCGTAGAGATGTGGAGGAACACCAGTGGCGAAGGCGGT

TTTTTGGTCTGTAACTGACGCTGAGGCGCGAAAGCGTGGGGAGCAAACAGGATTAGATACCCTGGTAGTCCACGCCGTAA

ACGATGAGTGCTAAGTGTTAGAGGGTTTCCGCCCTTTAGTGCTGCAGCTAACGCATTAAGCACTCCGCCTGGGGAGTACG

GTCGCAAGACTGAAACTCAAAGGAATTGACGGGGGCCCGCACAAGCGGTGGAACATGTTGTTTAATTCGAAGCAACGCGA

AGAACCTTACCAGGTCTTGACTTCCTCTGACAACTCTAAAAAAGAAACGTTCCCCTTCGGGGGACGAAATGACGGGGGGG

CATGGTTGT

>0424_894_012_PCR_AL_1_1492R_F10.ab1

CCGGGGGGCGGGGACCACATAAAGGGGCTAGCTCCTTAGAGTTACTCCACCGACTTCGGGTGTTACAAACTCTCGTGGTG

TGACGGGCGGTGTGTACAAGGCCCGGGAACGTATTCACCGCGGCATGCTGATCCGCGATTACTAGCGATTCCAGCTTCAT

GTAGGCGAGTTGCAGCCTACAATCCGAACTGAGAATGGTTTTATGGGATTGGCTTGACCTCGCGGTCTTGCAGCCCTTTG

TACCATCCATTGTAGCACGTGTGTAGCCCAGGTCATAAGGGGCATGATGATTTGACGTCATCCCCACCTTCCTCCGGTTT

GTCACCGGCAGTCACCTTAGAGTGCCCAACTAAATGCTGGCAACTAAGATCAAGGGTTGCGCTCGTTGCGGGACTTAACC

CAACATCTCACGACACGAGCTGACGACAACCATGCACCACCTGTCACTCTGTCCCCCGAAGGGGAACGCTCTATCTCTAG

AGTTGTCAGAGGATGTCAAGACCTGGTAAGGTTCTTCGCGTTGCTTCGAATTAAACCACATGCTCCACCGCTTGTGCGGG

CCCCCGTCAATTCCTTTGAGTTTCAGTCTTGCGACCGTACTCCCCAGGCGGAGTGCTTAATGCGTTAGCTGCAGCACTAA

AGGGCGGAAACCCTCTAACACTTAGCACTCATCGTTTACGGCGTGGACTACCAGGGTATCTAATCCTGTTTGCTCCCCAC

GCTTTCGCGCCTCAGCGTCAGTTACAGACCAAAAAGCCGCCTTCGCCACTGGTGTTCCTCCACATCTCTACGCATTTCAC

CGCTACACGTGGAATTCCGCTTTTCTCTTCTGCCCTCAAGTTCCCCAGTTTCCAATGACCCCTCCACGGTTGAGCCGTGG

GCTTTTCACTTCAAACTTAATAAAACCGCCTGCGCCCCCTTTACGCCCAATAATTTCCGGATAACCCCTTGCCCACCTAC

GAATTACCGCGGCTGCTGGCACGTAATTAACCCGTGGTTTTTTTGGTTAGGTACCGTCCAAGGAACAAACAA

AL-5

>0424_894_015_PCR_AL_5_27F_A11.ab1

AGGGGCGGCGGCTTAACACATGCAAGTCGAGCGGGGTGATGGTGCTTGCACTATCACTTAGCGGCGGACGGGTGAGTAAT

GCTTAGGAATCTGCCTATTAGTGGGGGACAACATCTCGAGAGGGATGCTAATACCGCATACGTCCTACGGGAGAAAGCAG

GGGATCACTTGTGACCTTGCGCTAATAGATGAGCCTAAGTCGGATTACCTAGTTGGTGGGGTAAAGGCCTACCAAGGCGA

CGATCTGTAGCGGGTCTGAGAGGATGATCCGCCACACTGGGACTGAGACACGGCCCAAACTCCTACGGGAGGCAGCAGTG

GGGAATATTGGACAATGGGGGGAACCCTGATCCAGCCATGCCGCGTGTGTGAAGAAGGCCTTATGGTTGTAAAGCACTTT

AAGCGAGGAGGAGGCTCTTTTGGTTAATACCCAAGATGAGTGGACGTTACTCGCAGAATAAGCACCGGCTAACTCTGTGC

CAGCACCCGCGGTAATACAGAGGGTGCAAGCGTTAATCGGATTTACTGGGCGTAAAGCGCGCGTAGGCGGCCAATTAAGT

CAAATGTGAAATCCCCGAGCTTAACTTGGGAATTGCATTCAATACTGGTTGGCTAGAGTGTGGGAGAGGATGGTAGAATT

CCAGGTGTAGCGGTGAGATGCGTAGAGATCTGGAGGAATACCGATGGCGAAGGCAGCCATCTGGCCTAACACTGACGCTG

AGGTGCGATAGCATGGGGAGCAAACAGGATTAAATACCCTGGTAGTCCATGCCGTACACGATGTCTACTAATCGTTGGGG

CCTTTGAGGCTTTATTGGCGCATCTAACGCGATAAGTACAACCGCCTGGGGTAGTACGGTCGCAAGACTAACACTCAAAT

GAATTGACGGGGGCCCGCACCAGCCGTGGAACATGTGGTTTAATTCGATGCAACTCCAAACAACCTTACCTGGCCTTGAC

CTAGGTAGAAACTTTCCAGAAAATGGATTGG

>0424_894_016_PCR_AL_5_1492R_B11.ab1

CAGTTCGGGGGGACCAGTAGGAGCGCGCCCTCTTTGCAGGATACGCTAGCTACTTCTGGTGCAACAAACTCCCATGGTGT

GACGGGCGGTGTGTACAAGGGCCGGGAACGTATTCACCGCGGCATTCTGATCCGCGATTACTAGCGATTCCGACTTCATG

GAGTCGAGTTGCAGACTCCAATCCGGACTACGATCGGCTTTTTGAGATTAGCATCCTATCGCTAGGTAGCAACCCTTTGT

ACCGACCATTGTAGCACGTGTGTAGCCCTGGCCGTAAGGGCCATGATGACTTGACGTCGTCCCCGCCTTCCTCCAGTTTG

TCACTGGCAGTATCCTTAAAGTTCCCATCCGAAATGCTGGCAAGTAAGGAAAAGGGTTGCGCTCGTTGCGGGACTTAACC

CAACATCTCACGACACGAGCTGACGACAGCCATGCAGCACCTGTATGTAGATTCCCGAAGGCACCAATCCATCTCTGGAA

AGTTTCTACTATGTCAAGGCCAGGTAAGGTTCTTCGCGTTGCATCGAATTAAACCACATGCTCCACCGCTTGTGCGGGCC

CCCGTCAATTCATTTGAGTTTTAGTCTTGCGACCGTACTCCCCAGGCGGTCTACTTATCGCGTTAGCTGCGCCACTAAAG

CCTCAAAGGCCCCAACGGCTAGTAGACATCGTTTACGGCATGGACTACCAGGGTATCTAATCCTGTTTGCTCCCCATGCT

TTCGCACCTCAGCGTCAGTGTTAGGCCAGATGGCTGCCTTCGCCATCGGTATTCCTCCAGATCTCTACGCATTTCACCGC

TACACCTGGGAATTCTACCATCCTCTCCCACACTCTAGCCACCAGTATCGAATGCAATTCCCAAGTTAAGCTCGGGGATT

TACATTTGACTTAATTGGCCGCCTACGCACGCTTTACGCCCAGTAAATCCAATAAACCCTTGCACCCTCTGTATTACCGC

GGGTGGTGGGCAGAAATTAGCCGGGGCTTATTCTGCGAGTAACGTCCACTGATCTTGGGTATTAACCAA

**AL-6**

>0424_894_017_PCR_AL_6_27F_C11.ab1

GGTGCCGGGACGGCGATAACGTGACACATGCAAGTCGAGACCGAAGTGTAGACCGATGACATTGCTCCCTGATTTAGCGG

CGGACGGGTGAGTAATGCCTAGGAATCTGCCTAGTGGTGGGGGATAACGTTCCGAAAGGAACGCTAATACCGCATACGTC

CTACGGGAGAAAGCGGGGGATCTTCGGACCTCGCGCCATTAGATGAGCCTAGGTCGGATTAGCTAGTTGGTGAGGTAAAG

GCTCACCAAGGCGACGATCCGTAACTGGTCTGAGAGGATGATCAGTCACACTGGAACTGAGACACGGTCCAGACTCCTAC

GGGAGGCAGCAGTGGGGAATATTGGACAATGGGCGAAAGCCTGATCCAGCCATGCCGCGTGTGTGAAGAAGGTCTTCGGA

TTGTAAAGCACTTTAAGTTGGGAGGAAGGGTATTCACCTAATACGTGAGTATTTTGACGTTACCGACAGAATAAGCACCG

GCTAACTTCGTGCCAGCAGCCGCGGTAATACGAAGGGTGCAAGCGTTAATCGGAATTACTGGGCGTAAAGCGCGCGTAGG

TGGTTCGTTAAGTTGGATGTGAAAGCCCCGGGCTCAACCTGGGAACTGCATCCAAAACTGGCGAGCTAGAGTACGGTAGA

GGGTGGTGGAATTTCCTGTGTAGCGGTGAAATGCGTAGATATAGGAAGGAACACCAGTGGCGAAGGCGACCACCTGGACT

GATACTGACACTGAGGTGCGAAAGCGTGGGGAGCAAACAGGATTAGATACCCTGGTAGTCCACGCCGTACACGATGTCAA

CTAGCCGTTGGAATCCTTGAGATTTTAGTGGCGCAGCTAACGCATTAAGTTGACCGCCTGGGGAGTACGGCCGCAAGGTT

AAAACTCAAATGAATTGACGGGGGCCCGCACAAGCGGGCGAGCATGGGGTTTAATTCGAAGCAACGCCAAGAAACCTTAC

CTGGCCTTGAATTGCTGAAAACTTTCCAGAAATGGATTGGGGGCCTTCCGGAAAATCAAAAACAGGGGCT

>0424_894_018_PCR_AL_6_1492R_D11.ab1

CTTTTGGGGGGGCCCGAACAGACTATGACGCTGCTCCAGCCAGAGGAGCATACACCTTAGCTACTTCTGGAGCAACCCAC

TCCCATGGTGTGACGGGCGGTGTGTACAAGGCCCGGGAACGTATTCACCGTGACATTCTGATTCACGATTACTAGCGATT

CCGACTTCACGCAGTCGAGTTGCAGACTGCGATCCGGACTACGATCGGTTTTATGGGATTAGCTCCACCTCGCGGCTTGG

CAACCCTTTGTACCGACCATTGTAGCACGTGTGTAGCCCTGGCCGTAAGGGCCATGATGACTTGACGTCATCCCCACCTT

CCTCCGGTTTGTCACCGGCAGTCTCCTTAGAGTGCCCACCATAACGTGCTGGTAACTAAGGACAAGGGTTGCGCTCGTTA

CGGGACTTAACCCAACATCTCACGACACGAGCTGACGACAGCCATGCAGCACCTGTGTCTGAGTTCCCGAAGGCACCAAT

CCATCTCTGGAAAGTTCTCAGCATGTCAAGGCCAGGTAAGGTTCTTCGCGTTGCTTCGAATTAAACCACATGCTCCACCG

CTTGTGCGGGCCCCCGTCAATTCATTTGAGTTTTAACCTTGCGGCCGTACTCCCCAGGCGGTCAACTTAATGCGTTAGCT

GCGCCACTAAAATCTCAAGGATTCCAACGGCTAGTTGACATCGTTTACGGCGTGGACTACCAGGGTATCTAATCCTGTTT

GCTCCCCACGCTTTCGCACCTCAGTGTCAGTATCAGTCCAGGTGGTCGCCTTCGCCACTGGTGTTCCTTCCTATATCTAC

GCATTTCACCGCTACACAGGGAAATTCCACCACCCTCTACCGTACTCTAGCTCGCCAGTTTTTGGATGCAGTTCCCAGGT

TGAACCCCGGGGCTTTCACATCCAACTTAACGAAACCACCTAACGCGCGCTGTTACGCCCAGAAATTACCAATTAACCCT

TGCACCCTTCCTATTTACCGCGGCTGCTTGGCACGAAAGTTAGCCGGGGCTTTTTTTTGTCGGGAAACGTCAAAATAACT

CACCTTTTAGGGGGAAAAACCCCTCCCTCCCCAACTTAAAGA

**AL-9**

>0424_894_019_PCR_AL_9_27F_E11.ab1

GGGCGTGGCGGGCGAGCTTACACATGCAAGTCGAGCCAGAGTGATCGCGTGACATTGCACTATCACTTAGCGGCGGACGG

GTGAGTAATGCTTAGGAATCTGCCTATTAGTGGGGGACAACATCTCGAAAGGGATGCTAATACCGCATACGTCCTACGGG

AGAAAGCAGGGGATCACTTGTGACCTTGCGCTAATAGATGAGCCTAAGTCGGATTAGCTAGTTGGTGGGGTAAAGGCCTA

CCAAGGCGACGATCTGTAGCGGGTCTGAGAGGATGATCCGCCACACTGGGACTGAGACACGGCCCAGACTCCTACGGGAG

GCAGCAGTGGGGAATATTGGACAATGGGGGGAACCCTGATCCAGCCATGCCGCGTGTGTGAAGAAGGCCTTATGGTTGTA

AAGCACTTTAAGCGAGGAGGAGGCTCTTTTGGTTAATACCCAAGATGAGTGGACGTTACTCGCAGAATAAGCACCGGCTA

ACTCTGTGCCAGCAGCCGCGGTAATACAGAGGGTGCAAGCGTTAATCGGATTTACTGGGCGTAAAGCGCGCGTAGGCGGC

CAATTAAGTCAAATGTGAAATCCCCGAGCTTAACTTGGGAATTGCATTCGATACTGGTTGGCTAGAGTGTGGGAGAGGAT

GGTAGAATTCCAGGTGTAGCGGTGAAATGCGTAAAGATCTGGAGGAATACCGATGGCGAAGGCAGCCATCTGGCCTAACA

CTGACGCTGAGGTGCGAAAGCATGGGGAGCAAACAGGATTAGATACCCTGGTAGTCCATGCCGTAAACGATGTCTACTAG

CCGTTGGGGTCCTTTGAGGCTTTAGTGGCGCAGCTAACGCGATAAGTAAACCGCCTGGGGAGTACGGTCGCAAGACTAAA

ACTCAGATGAATTGACGGGGGCCCGCACAACCGGTGGAGCATGTGGTTTAATTCGATGCAACGCAAAAGAACCTTACCTG

GCCTTGACATAGAAGAAAACTTTCCAAAAATGGATTGGTGCCTTCGGGAATCTAAATACAGGTGCT

>0424_894_020_PCR_AL_9_1492R_F11.ab1

GCCGGGGTTCATCGAAGACCACCGATGTGTGAACCGCCCTCTCATGCATGCATACGCTAGCTACTTCTGGTGCAACAAAC

TCCCATGGTGTGACGGGCGGTGTGTACAAGGCCCGGGAACGTATTCACCGCGGCATTCTGATCCGCGATTACTAGCGATT

CCGACTTCATGGAGTCGAGTTGCAGACTCCAATCCGGACTACGATCGGCTTTTTGAGATTAGCATCCTATCGCTAGGTAG

CAACCCTTTGTACCGACCATTGTAGCACGTGTGTAGCCCTGGCCGTAAGGGCCATGATGACTTGACGTCGTCCCCGCCTT

CCTCCAGTTTGTCACTGGCAGTATCCTTAAAGTTCCCATCCGAAATGCTGGCAAGTAAGGAAAAGGGTTGCGCTCGTTGC

GGGACTTAACCCAACATCTCACGACACGAGCTGACGACAGCCATGCAGCACCTGTATGTAGATTCCCGAAGGCACCAATC

CATCTCTGGAAAGTTTCTACTATGTCAAGGCCAGGTAAGGTTCTTCGCGTTGCATCGAATTAAACCACATGCTCCACCGC

TTGTGCGGGCCCCCGTCAATTCATTTGAGTTTTAGTCTTGCGACCGTACTCCCCAGGCGGTCTACTTATCGCGTTAGCTG

CGCCACTAAAGCCTCAAAGGCCCCAACGGCTAGTAGACATCGTTTACGGCATGGACTACCAGGGTATCTAATCCTGTTTG

CTCCCCATGCTTTCGCACCTCAGCGTCAGTGTTAGGCCAGATGGCTGCCTTCGCCATCGGTATTCCTCCAGATCTCTACG

CATTTCACCGCTACACCTGGAATTCTACCATCCTCTCCCACACTCTAGCCAACCAGTATCGAATGCAATTCCCAAGTTAA

GCTCGGGGATTTCACATTTGACTTAATTGGCCGCCTACGCGCGCTTTACGCCCAGTAAATCCGATTAACGCTTGCACCCT

CTGTATTACCGCGGCTGCTGGCACAAAGTTAACCGGTGCTTATTCTGCAAGAAACGTCCACTCATCTTGGGGATTAACCA

AAAGAACCTCCTCCTCGCTTAAAGGGTTTTCAACCCTAAAGGCCTTCTTC
